# Methadone for critically ill patients under mechanical ventilation in the intensive care unit: a systematic review

**DOI:** 10.62675/2965-2774.20250396

**Published:** 2025-07-15

**Authors:** Sérgio Martins Pereira, Megan Abbott, João Francisco Figueiredo Marcondes Ferraz, Akash Goel, Andrea Rigamonti, Charmaine de Castro, Lisa Burry, Airton Leonardo de Oliveira Manoel, Michael Chaim Sklar

**Affiliations:** 1 University of Toronto Department of Anesthesiology and Pain Medicine Toronto Ontario Canada Department of Anesthesiology and Pain Medicine, University of Toronto - Toronto, Ontario, Canada.; 2 University of Toronto Temerty Faculty of Medicine Toronto Ontario Canada Temerty Faculty of Medicine, University of Toronto - Toronto, Ontario, Canada.; 3 Baylor College of Medicine Section of Pulmonary, Critical Care, and Sleep Medicine Houston Texas United States Section of Pulmonary, Critical Care, and Sleep Medicine - Baylor College of Medicine - Houston, Texas, United States.; 4 University of Toronto Interdepartmental Division of Critical Care Medicine Toronto Ontario Canada Interdepartmental Division of Critical Care Medicine, University of Toronto - Toronto, Ontario, Canada.; 5 Sinai Health Mount Sinai Hospital Sidney Liswood Library Toronto Ontario Canada Sidney Liswood Library, Mount Sinai Hospital, Sinai Health - Toronto, Ontario, Canada.; 6 Sinai Health Departments of Pharmacy and Medicine Toronto Ontario Canada Departments of Pharmacy and Medicine, Sinai Health - Toronto, Ontario, Canada.; 7 Sultan Qaboos Comprehensive Cancer Care and Research Centre Department of Intensive Care Medicine Muscat Oman Department of Intensive Care Medicine, Sultan Qaboos Comprehensive Cancer Care and Research Centre - Muscat, Oman.

**Keywords:** Methadone, Respiration, artificial, Opioid, Analgesia, Sedation

## Abstract

**Purpose:**

Pain may pose significant challenges in the intensive care unit, especially in mechanically ventilated patients. Methadone has recently emerged as an alternative option for eliciting acute analgesia. In this systematic review, we evaluated the use of methadone in mechanically ventilated patients in the intensive care unit.

**Source:**

We searched MEDLINE, EMBASE, Wiley's Cochrane Library, CINAHL, PubMed (non-MEDLINE), Scopus, and LILACS databases from inception to January 24^th^, 2025. Eligible studies included randomized controlled trials and observational studies that compared the use of methadone to the standard of care or to other analgosedation strategies in mechanically ventilated patients in the intensive care unit. The primary outcome was the duration of mechanical ventilation. The secondary outcomes included opioid-associated adverse effects and scores regarding pain, agitation, and *delirium*.

**Principal:**

findings: The search strategy yielded 3,523 studies. A total of 773 patients were included across the 12 studies (including 7 abstracts and 5 manuscripts). Patient populations included patients with trauma, those with burns, those at high risk for fentanyl abstinence syndrome, those with opioid use disorder, those with opioid withdrawal symptoms, and those who had received fentanyl for 72 hours prior to weaning. Overall, compared with the group that did not receive methadone, the methadone group was associated with more ventilator-free days, shorter weaning times, and a greater probability of successful weaning on day 5. Most of the studies exhibited high risks of bias; moreover, the overall quality of the evidence was low.

**Conclusion:**

Few studies have evaluated the use of methadone in mechanically ventilated patients. Based on the low-quality evidence, methadone may be associated with improved patient-centered outcomes. Further research is warranted with respect to this topic.

## INTRODUCTION

Pain and discomfort are pervasive challenges encountered in the intensive care unit (ICU).^(1,2)^ In mechanically ventilated patients, inadequate pain management often manifests as psychomotor agitation and patient-ventilator asynchrony,^(3)^ which may be mitigated via careful titration of analgesia and/or sedation.^(3)^ A recent study revealed that moderate to deep sedation significantly prolongs ventilator weaning and leads to adverse outcomes,^(4)^ thus highlighting the need for targeted strategies to address these challenges.

Opioids induce systemic analgesia, anxiolysis, and sedation; additionally, they are commonly administered to ventilated patients^(5,6)^ to manage the discomfort associated with the insertion of an endotracheal tube or the performance of certain procedures. Methadone is a long-acting μ-opioid receptor agonist that exhibits additional agonist activity at the κ- and σ-opioid receptors. In addition to the inhibition of monoamine and catecholamine reuptake, the effect of methadone on the N-methyl-aspartate-D-aspartate (NMDA) receptor has been postulated to elicit analgesia and euphoria while preventing the development of acute opioid tolerance.^(7,8)^ Although potentially beneficial, only one randomized clinical trial (RCT) has evaluated methadone in mechanically ventilated patients in the ICU.^(9)^

Although there is growing adoption of methadone in the operating room setting,^(10,11)^ limited data on other settings exist. Current clinical practice guidelines highlight the importance of addressing the risks of opioid withdrawal and opioid use disorders when managing ICU patients,^(3)^ scenarios in which methadone is a cornerstone treatment. Based on these considerations, we performed a systematic review to evaluate studies in which methadone was administered to mechanically ventilated patients in the ICU and to understand the effects of methadone on mechanical ventilation (MV)-related outcomes, total opioid exposure, associated complications, and scores regarding pain, sedation, and *delirium*.

## METHODS

### Design

We conducted a systematic review with predetermined selection and outcome criteria following the Cochrane Handbook,^(12)^ and this review was registered on PROSPERO (registration: CRD42024517731). Prior to the comprehensive literature search, we searched for ongoing systematic reviews on PROSPERO. Afterwards, an information specialist designed a comprehensive literature search strategy. We searched the following databases from inception to January 24, 2025: MEDLINE, EMBASE, Wiley's Cochrane Library, CINAHL, PubMed (non-MEDLINE), Scopus, and LILACS. Eligible studies included RCTs and observational studies that compared the use of methadone to the standard of care or to other analgosedation strategies in mechanically ventilated patients in the ICU. Additionally, we searched the Clinical Trials Registry Database (https://clinicaltrials.gov) for registered, unpublished, and ongoing studies. We also searched the bibliographies of the included studies and review articles. Studies were restricted to English, Portuguese, and Spanish languages (Supplementary Material).

We included studies that described adult (over 18 years of age) patients receiving MV in the ICU who had also received methadone. In our initial design, pain, sedation, and *delirium* scores were our main outcomes. During screening and data extraction, we observed that most of the studies focused on MV-related outcomes; therefore, we changed the main outcomes to MV-related outcomes, which included the duration of MV, ventilator-free days, successful weaning, and weaning time. The secondary outcomes included total opioid exposure; incidences of nausea and vomiting, coma, withdrawal to opioids and arrhythmias; ICU and hospital lengths of stay; incidence of hospital-acquired pneumonia; time to weaning from intravenous or oral sedation; and scores regarding pain, sedation, and *delirium*. Pain and sedation scores were assessed in the included studies via behavioral pain scales,^(13)^ visual analog scales or numeric rating scales; sedation scores were assessed in the studies via the Richmond Analgesia and Sedation Scale (RASS),^(14)^ the Ramsay Sedation Scale,^(15)^ and the Riker Sedation-Agitation Scale;^(16)^ and *delirium* was assessed in the studies via the Confusion Assessment Method for the ICU.^(17)^ We reported our review based on the PRISMA guidelines.^(18)^

### Control and intervention groups

The intervention group included patients who received methadone (either alone or in addition to other opioids) as a strategy of sedation. The control group included patients receiving the standard-of-care strategy of sedation without methadone.

### Study selection

Two authors independently reviewed abstracts and assessed eligibility via Covidence systematic review software (Veritas Health Innovation, Melbourne, Australia, 2024). A full-text review was conducted when either reviewer considered that the abstract met the inclusion criteria. Both reviewers agreed on the full text for inclusion, with an independent reviewer resolving any disagreements.

### Data extraction and synthesis

All of the data were extracted in duplicate. The authors independently extracted the following data from the included studies: study and patient characteristics, study interventions, total opioid use, and outcomes. Both reviewers independently assessed the risk of bias at the outcome level regarding randomization, deviations from intended interventions, missing outcome data, and selection of the reported result. The Cochrane risk of bias tool for randomized studies (RoB2)^(19)^ and the risk of bias in nonrandomized studies of interventions (ROBINS-I) tool^(20)^ were used to assess the risks of bias in randomized and nonrandomized studies, respectively. The third reviewer resolved any discrepancy that arose during this assessment. Additionally, the quality of evidence for each manuscript was assessed by the reviewers via the GRADE framework and is reported by outcome.^(21)^

## RESULTS

### Study selection

**Figure 1:**
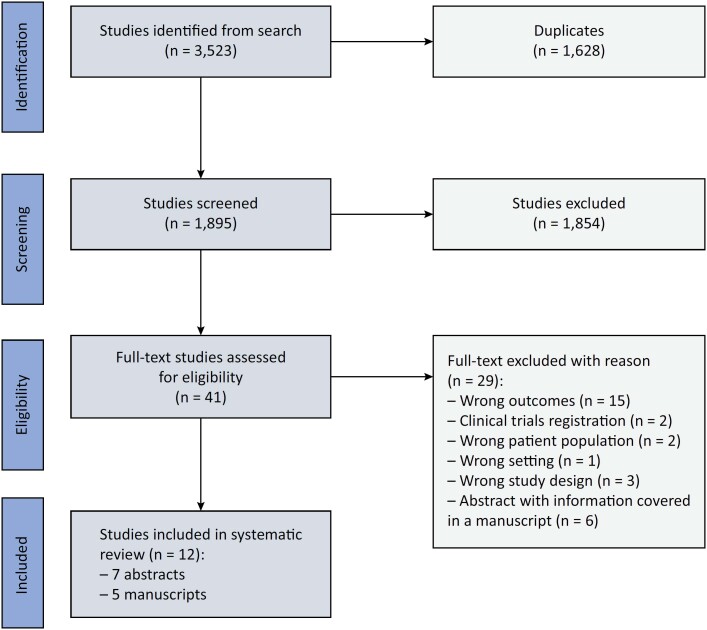
Identification of studies from the search strategy based on the inclusion and exclusion criteria.

The search strategy yielded 3,523 citations, of which 1,628 were duplicates. The remaining 1,895 abstracts were screened, with 1,568 being excluded. We completed a full-text review of 38 studies, of which 12 studies met the inclusion criteria, including 7 abstracts^(22-28)^ and 5 manuscripts^(9,29-32)^ (Figure 1).

### Characteristics of the included studies

Table 1 summarizes the study type, population, control, intervention, and outcomes of the included studies. A total of 773 patients were included among the included abstracts and manuscripts. All of the included studies were generally small in size, with sample sizes ranging from 16 to 118 patients. The included studies had heterogeneous designs, methadone dosages, and patient populations. The characteristics of the patients included trauma (n = 118),^(22)^ burn injuries (n = 153),^(24,29)^ risk for fentanyl abstinence syndrome (n = 84),^(23,26)^ opioid addiction or withdrawal (n = 106),^(25,32)^ and receiving fentanyl for at least 72 hours prior to weaning (n = 221).^(9,23,26,31)^ The administration of methadone was compared to the nonadministration of methadone,^(22,27)^ standard of care (n = 213),^(24,29,31)^ fentanyl administration (n = 140),^(23,26,32)^ opium tincture administration,^(25)^ oxycodone administration,^(30)^ and placebo administration.^(9)^ Standard of care and the nonadministration of methadone were defined as the groups of patients who did not receive methadone; moreover, opium tincture is a medication that is commonly used to treat diarrhea and may be used as an alternative to methadone when treating patients with opioid use disorders.

**Table 1:** Included abstracts and manuscripts

Author, journal and year	Study type	Population	Control	Intervention	Outcomes
Wanzuita et al.^(9)^ Critical Care, 2012 *Manuscript*	Randomized controlled trial	Patients weaning from MV who had been using fentanyl for more than 5 consecutive days	Placebo N = 31 Age = 45 ± 17 Male = 26 (83)	Methadone PO N = 37 Age = 43 ± 18 Male = 27 (72)	1) Probability of successful weaning by the fifth day was higher in methadone patients: HR: 2.64 (1.22 to 5.69); p < 0.02 2) Higher probability of early extubation with methadone: HR: 1.52 (0.87 to 2.64); p = 0.11 3) Among patients who were successfully weaned, the MV weaning time was lower in the methadone group: HR: 2.06 (1.17 to 3.63); p < 0.004
Kowalski et al.^(28)^* Critical Care Medicine, 2018 *Abstract*	Retrospective cohort	MV for 48 hours	Immediate release opioids	Methadone	1) Time to wean continuous infusion: 57.5 *versus* 69 hours, p = 0.527 2) QTc: 460 *versus* 455ms; p = 0.11 3) Long-acting opioid prescription following ICU discharge: 2.7% *versus* 51.4%; p< 0.001
Jones et al. ^(22)^ Critical Care Medicine, 2011 *Abstract*	Retrospective cohort with propensity match score	Patients who received methadone within 4 days of intubation and who remained ventilated for 2 days after the first dose	No methadone N = 88	Early methadone N = 30	1) Ventilator-free days: 12.6 *versus* 15.2; p = 0.18 2) Incidence of VAP: 39.8% *versus* 20%; p = 0.05
Zavala et al.^(24)^ Journal of Burn Care and Research, 2021 *Abstract*	Retrospective cohort	Adult patients admitted to the burn center for initial management who required MV for at least 48 hours, without prior methadone use and total body surface area > 5%	No methadone N = 31 Age = 56.2	Methadone N = 52 Age = 45.3	1) Ventilator-free days: 15 *versus* 9.5; p = 0.009 2) ICU LOS: 35.8 *versus* 57.2 days; p = 0.025 3) Duration of analgesia: 12 *versus* 20.7 days; p = 0.011 4) Duration of sedation: 12.4 *versus* 19 days; p=0.026
Maghsoudi^(25)^ Intensive Care Medicine Experimental, 2016 *Abstract*	Randomized pilot study	Inhalant opium-addicted ICU patients	Opium tincture N = 25	Methadone N = 25	1) BPS: benefit in opium tincture group; p < 0.019 2) RASS: no difference; p < 0.630
Wanzuita et al.^(26)^ Critical Care, 2011 *Abstract*	Prospective cohort	Patients fulfilling criteria for weaning from MV but under high risk for fentanyl abstinence syndrome (continuous fentanyl > 5 days or more than 5mcg/kg/hour during 12 hours)	Enteral placebo + IV fentanyl N = 31	Enteral methadone + IV placebo N = 37	1) Anticipation of extubation: HR: 1.44 (0.81 to 2.56); p = 0.21 2) Probability of successful weaning on the 5th day: 2.27x greater in methadone group, 13.28 ± 12.85 days; p < 0.004
Bonnin et al.^(27)^ Critical Care Medicine, 2020 *Abstract*	Retrospective cohort	Patients on continuous opioid infusion for a minimum of 24 hours	No methadone N = 8	Enteral methadone N = 9	1) Cumulative dose in MME: 1,596 [805 - 2,359] *versus* 1,778 [1,573 - 2,080]; p = 0.81 2) ICU LOS: 10 *versus* 11.9 days; p = 0.471 3) Incidence of *delirium*: 87.5% *versus* 88.9% 4) Incidence of increased QTc from baseline (≥ 60ms): 12.5% *versus* 11.1%
Wanzuita et al.^(23)^ Critical Care, 2007 *Abstract*	Randomized controlled trial	Patients under high risk for fentanyl abstinence syndrome (continuous fentanyl > 5 days or more than 5mcg/kg/hour during 12 hours)	Enteral placebo + IV fentanyl N = 9	Enteral methadone + IV placebo N = 7	1) ICU LOS: 27 (13) *versus* 13 (3) days; p < 0.02 2) Days under MV: 20 (21) *versus* 4 (0.8) days; p < 0.05
Jones et al.^(29)^ Journal of Burn Care & Research, 2013 *Manuscript*	Retrospective cohort with propensity match score	Patients between 18 and 89 years of age who required ventilation for at least 2 days	Standard of care N = 52 Age = 52.4 ± 17.6 Male = 38 (73.1)	Methadone PO/IV N = 18 Age = 43.4 ± 12.1 Male = 11 (61.1)	1) Ventilator-free days: 11.5 *versus* 16.5; p = 0.03 2) Patients on methadone more likely to be discharged alive: 88.9% *versus* 67.3%; p = 0.04 3) ICU LOS: 14 [6 - 26] *versus* 17 [8 - 27]; p = 0.74 4) Hospital LOS: 21 [11 - 34] *versus* 31 [21 - 50] days; p = 0.12 5) Incidence of VAP: 36.5% *versus* 27.8%; p = 0.37
Azimi et al.^(30)^ Annals of Pharmacotherapy, 2023 *Manuscript*	Retrospective cohort	Patients who received fentanyl or hydromorphone continuously for ≥ 72 hours	Oxycodone PO N = 57 Age = 50 ± 16 Male = 28 (49.1)	Methadone PO N = 36 Age = 49.7 ± 17 Male = 20 (55.6)	1) Time to discontinuation of IV opioid infusions from the initiation of enteral opioids: 158.3 ± 171.2 *versus* 104.7 ± 79.4; p = 0.04 2) Patients receiving methadone were 1.89x more likely to be weaned from the ventilator than oxycodone: HR: 1.89 (1.16 to 3.07; p = 0.01)
Al-Qadheeb et al.^(31)^ Annals of Pharmacotherapy, 2012 *Manuscript*	Prospective matched cohort 1:2	Patients without chronic opioid use who received ≥ 72 hours of continuously infused fentanyl	Standard of care N = 40 Age = 59.4 ± 14.2 Male = 13 (65)	Methadone PO/IV N = 20 Age = 42.7 ± 16 Male = 13 (65)	1) Time to fentanyl discontinuation 7.0 (4.9 to 11.5) *versus* 4.5 (3.9 to 5.8); p = 0.002 2) Fentanyl was more likely to be discontinued after methadone was initiated: HR: 3.8 (1.7 to 8.8); p = 0.001
Najafi et al.^(32)^ Archives of Academic Emergency Medicine, 2021 *Manuscript*	Prospective controlled pilot study	Opioid-dependent patients admitted to the toxicology ICU after initiation of their withdrawal syndrome	Fentanyl N = 28 Age = 44 (22 to 57)	Methadone N = 28 Age = 41 (26 to 55)	1) Duration of intubation: 5 [5 - 50] *versus* 9 [4 -17] days; p = 0.120 2) ICU LOS: 7 [6 - 14] *versus* 10 [7 - 14] days; p = 0.572 3) Withdrawal symptoms after administration: 120 [45 - 120] *versus* 30 [30 - 60] minutes; p = 0.008 4) COWS: time of withdrawal: 18 *versus* 18; p = 0.537; 30 minutes after administration: 6 *versus* 6; p = 0.967; 120 minutes after administration: 0 *versus* 2; p = 0.136

MV - mechanical ventilation; PO - per oral; HR - hazard ratio; QTc - corrected QT interval in an electrocardiogram; ICU - intensive care unit; VAP - ventilator-associated pneumonia; LOS - length of stay; BPS - Behavioral Pain Scale; RASS - Richmond Analgesia and Sedation Scale; IV - intravenous; MME - milligrams morphine equivalents; COWS - Clinical Opiate Withdrawal Scale. All of the data are presented as control *versus* intervention. Some studies did not describe the age and gender of their sample. All of the outcomes are control versus intervention.

* Total of 74 patients. The study did not report the sample size of each group. Data are described as the means ± standard deviations, median [interquartile range)] or n (%).

### Mechanical ventilation-related outcomes

Mechanical ventilation-related outcomes were reported (either as a primary or secondary outcome) in all but two of the studies.^(25,31)^ The included studies described ventilator-free days (n = 271),^(22,24,29)^ days on MV,^(23)^ anticipation of extubation,^(26)^ duration of intubation,^(26)^ successful weaning,^(30)^ weaning success on day 5 (n = 136),^(9,26)^ early extubation,^(26)^ and decreased weaning time.^(9)^

One RCT^(9)^ revealed that the enteral administration of methadone led to a significantly greater probability of successful weaning within the first five days (hazard ratio [HR]: 2.64 [1.22 to 5.69]; p < 0.02), as did a retrospective cohort (HR: 1.89 [1.16 to 3.07]; p = 0.01]).^(30)^ These results were corroborated by two abstracts from the same group of investigators.^(23,26)^ Enteral methadone use was also associated with a shorter weaning time (HR: 2.06 [1.17 to 3.63]; p < 0.004) but not with the probability of early extubation (HR: 1.52 [0.87 to 2.64]; p = 0.11).^(9)^ In terms of ventilator-free days, a pilot study^(29)^ and a retrospective chart review^(24)^ indicated that methadone was associated with significantly more ventilator-free days (11.5 days *versus* 16.5 days; p = 0.03 and 15 days *versus* 9.5 days; p = 0.009, respectively), whereas a propensity score-matched cohort observed no such benefit (12.6 *versus* 15.2; p = 0.18).^(22)^

### Secondary outcomes

The secondary outcomes included length of stay in the ICU (n = 172),^(23,27,30,32)^ development of ventilator-associated pneumonia,^(22)^ duration of analgesia,^(24)^ cumulative dose of opioids in morphine equivalents,^(27)^ duration of sedation,^(24)^ behavior pain scale,^(25)^ incidence of *delirium*,^(27)^ RASS score,^(25)^ patients discharged alive,^(29)^ adverse effects from methadone administration,^(27)^ discontinuation of opioids (n = 227),^(28,30,31)^ withdrawal symptoms,^(32)^ and Clinical Opiate Withdrawal Score (COWS) score.^(32)^

The use of methadone to discontinue opioid infusions was evaluated in three studies. One study^(31)^ reported that enteral methadone administration shortened the time needed to discontinue fentanyl infusions in critically ill patients (4.5 [3.9 to 5.8] days *versus* 7.0 [4.9 to 11.5] days; p = 0.002), which is similar to the results of Azimi et al.,^(30)^ who reported that IV opioid discontinuation was faster with methadone than with oxycodone (104.7 ± 79.4 hours *versus* 158.3 ± 171.2 hours, respectively; p = 0.04). Conversely, in another study, methadone was not observed to be associated with a reduction in the time to weaning for continuous infusion (57.5 hours *versus* 69 hours; p = 0.527).^(28)^ Bonnin et al.^(27)^ did not identify any evidence of any effects of enteral methadone administration on total opioid intake, ICU length of stay, incidence of *delirium* or increased QTc from a baseline cumulative value. Moreover, methadone provided faster alleviation of withdrawal symptoms compared to fentanyl, with symptoms subsiding within 30 minutes for methadone *versus* 120 minutes for fentanyl (p = 0.007).^(32)^ Both drugs effectively controlled withdrawal symptoms over time, with no significant differences in ICU or hospital stay duration, duration of intubation, or complications being observed between the groups.

### Methodological quality of the included studies

**Figure 2:**
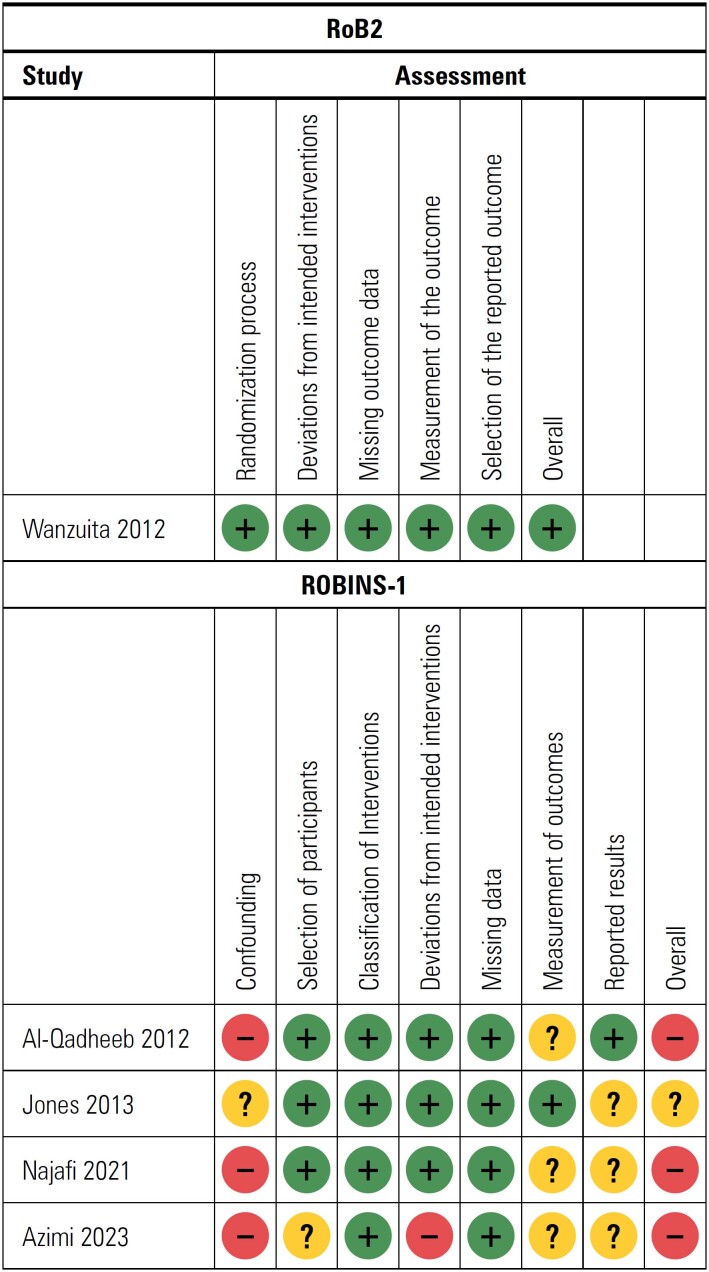
Risk of bias summary

A summary of evidence is outlined in table 2 for each outcome that was assessed in the manuscripts included in our review. For the primary outcome, only one study demonstrated high-quality data with a low risk of bias.^(9)^ The other two studies^(29,32)^ demonstrated low-to-moderate quality data, as well as a moderate-to-serious risk of bias. The overall effect of methadone was either likely beneficial^(9,29)^ or had no effect.^(32)^ For secondary outcomes including ICU outcomes, opioid discontinuation, and opioid withdrawal symptoms, the administration of methadone was either likely beneficial or had no effects, although this was based on low-quality evidence and a high risk of bias. The risk of bias summary is presented in figure 2, and the GRADE quality of the evidence assessment per study is presented in table 3.

**Table 2:** Summary of the GRADE quality of evidence assessment for the complete studies included in this systematic review and overall quality of evidence designation

GRADE assessment and risk of bias						
Primary outcome	Group		Outcome measured (source; units)	GRADE	Risk of bias	Overall effect
	Intervention	Control				
Mechanical ventilation-related	16.5	11.5	Ventilator-free days, days^(29)^	Moderate	Moderate	Likely beneficial
	HR: 1.89 (1.16 to 3.07)		Probability to be weaned from the ventilator, HR^(30)^	Moderate	Moderate	Likely beneficial
	HR: 2.64 (1.22 to 5.69)		Probability of successful weaning by the fifth day, HR^(9)^	High	Low	Likely beneficial
	HR: 1.52 (0.87 to 2.64)		Probability of early extubation, HR^(9)^	High	Low	Likely beneficial
	HR: 2.06 (1.17 to 3.63)		MV weaning time was lower in the methadone group among patients who were successfully weaned^(9)^	High	Low	Likely beneficial
	9 [4 - 17]	5 [5 - 50]	Duration of intubation, days^(32)^	Low	Serious	No effect
ICU outcomes	88.9%	67.3%	% of patients discharged alive from ICU^(29)^	Moderate	Moderate	Likely beneficial
	10 [7 - 14]	7 [6 - 14]	Duration of ICU stay, days^(32)^	Low	Serious	No effect
Other opioid discontinuation	104.7 ± 79.4	158.3 ± 171.2	IV opioid infusion, hours^(30)^	Moderate	Moderate	Likely beneficial
	4.5 (3.9 to 5.8)	7.0 (4.9 to 11.5)	Time to fentanyl discontinuation, days^(31)^	Moderate	Moderate	Likely beneficial
	HR: 3.8 (1.7 to 8.8)		Odds to discontinue fentanyl after methadone was initiated^(31)^	Moderate	Moderate	Likely beneficial
Opioid withdrawal	30 [30 - 60]	120 [45 - 120]	Symptoms after drug administration, minutes^(32)^	Low	Serious	No effect
	6 [1 - 21]	6 [4 - 13]	COWS 30 minutes after administration^(32)^	Low	Serious	No effect
	2 [1 - 3]	0 [0 - 3]	COWS 120 minutes after administration^(32)^	Low	Serious	No effect

HR - hazard ratio; MV - mechanical ventilation; ICU - intensive care unit; IV - intravenous; COWS - Clinical Opiate Withdrawal Scale.

RoB-2 - risk of bias in randomized trials; ROBINS-I - risk of bias in nonrandomized studies of interventions.

Green: low risk of bias; yellow: moderate risk of bias; red: high risk of bias.

**Table 3:** GRADE quality of evidence assessment per study

Study, year	Study design	Risk of bias	Inconsistency of results	Indirectness of evidence	Imprecision	Publication bias	Quality of evidence for individual study (n = 1)
Wanzuita et al.^(9)^ 2012	Randomized trial	Low risk	Not serious	Not serious	Not serious	Undetected	High ⊕⊕⊕⊕
Jones et al.^(29)^ 2013	Retrospective cohort with propensity match score	Unclear risk	Serious risk	Not serious	Not serious	Undetected	Moderate ⊕⊕⊕
Azimi et al.^(30)^ 2023	Retrospective cohort	Unclear risk	Serious risk	Not serious	Not serious	Undetected	Moderate ⊕⊕⊕
Al-Qadheeb et al.^(31)^ 2012	Prospective matched cohort	Unclear risk	Serious risk	Not serious	Not serious	Undetected	Moderate ⊕⊕⊕
Najafi et al.^(32)^ 2021	Prospective pilot	High risk	Serious risk	Serious risk	Serious	Undetected	Low ⊕⊕

## DISCUSSION

We summarized the current literature on methadone administration in mechanically ventilated patients in the ICU. Overall, in low-quality studies with a high risk of bias, the use of methadone was associated with improved ventilation-associated outcomes and faster opioid infusion discontinuation, although without benefits for managing withdrawal symptoms.

Long-term opioid use can lead to physical dependence, which places 16 - 32% of adult patients at risk for withdrawal syndrome in the ICU.^(33)^ The clinical manifestations of withdrawal syndrome overlap with the criteria assessed in patients who are weaning from the ventilator,^(34)^ thereby potentially postponing extubation. Moreover, the evaluation and management of opioid withdrawal may be challenging.^(33,35)^ Alternative strategies, such as alpha_2_-adrenergic agonists, are associated with more adverse effects compared to methadone.^(36)^ Interestingly, methadone exerts benefits for infants who require prolonged MV;^(37)^ moreover, it is the standard of care for children who are intubated for acute respiratory failure^(38)^ and effectively minimizes opioid withdrawal symptoms in at-risk children.^(39)^ Conversely, in adults with chronic opioid use disorder, methadone use was not observed to be associated with improvements in the RASS or COWS score. At least two factors may explain these discrepancies: (1) the RASS and COWS are unsuitable for assessing opioid withdrawal in intubated patients, which may have biased the results;^(40)^ and (2) inappropriate opioid conversion led to differing dosages between the groups, thereby leading to difficulties in attributing these findings solely to the medications (rather than to the dosing discrepancies). As a result, the effectiveness of methadone administration for opioid withdrawal in adults remains uncertain.

Methadone may also be employed to facilitate opioid tapering, which involves careful adjustments and/or opioid conversion. In the pediatric population, methadone has been suggested for use over other opioid regimens;^(41)^ however, this finding was not corroborated in a meta-analysis.^(42)^ In adults, methadone was observed to be associated with shorter opioid discontinuation times when compared to standard care^(31)^ or other opioids.^(30)^ Notably, pharmacokinetic-dynamic differences exist between methadone and shorter-acting opioids. For example, fentanyl infusion may exhibit an increased context-sensitive half-life^(43)^, whereas the active metabolites of morphine can paradoxically result in hyperalgesia.^(44)^ Conversely, methadone binds to multiple receptors, thereby functioning as a single-drug multimodal analgesic approach.^(45)^ We questioned whether the improved outcomes were secondary to improved pain management, thus leading to less agitation and an increased likelihood of extubation. Unfortunately, none of the studies measured metabolite serum concentrations.

Finally, ICU patients develop neuroplastic changes that contribute to pain^(46)^ and are often treated with opioids.^(47,48)^ However, the benefits of methadone may be limited to patients using high-dose opioids. For example, in the study by Wanzuita et al.,^(9)^ the mean dose of morphine in milligram equivalents was 422mg/day in the methadone group, which was 4 - 8 times greater than that reported by Burry et al.^(48)^ but was half that reported by Payen et al.^(13)^ It is possible that high-dose opioid use is secondary to an analgesia-first approach to achieve appropriate sedation goals, thus leading to opioid misuse. Strategies to minimize opioid use include multimodal analgesia, opioid rotation, and opioid weaning protocols, all of which represent indications for methadone.

### Clinical implications and considerations

The applicability of methadone to every intubated patient is not recommended, given that most studies only included patients who were at risk for opioid withdrawal. We suggest adhering to the approach by Wanzuita et al. (the only RCT included in our review).^(9)^ Although intravenous methadone administration offers more reliable bioavailability compared to enteral administration,^(49)^ its use should be approached with caution due to a lack of supporting evidence.

However, the administration of methadone may be associated with adverse cardiac effects.^(50)^ A prolonged QT interval is a surrogate marker for developing Torsades de Pointes, which is a rare and lethal ventricular arrhythmia.^(51)^ QT interval prolongation has been observed to occur when methadone is used at doses higher than 60 - 120mg/day,^(52,53)^ which is a limit that was not exceeded in this review. In addition, inappropriate methadone dosing may impair the function of vital systems, thereby significantly impacting patient outcomes. For example, high doses of methadone may lead to an overly sedated patient who may not trigger the ventilator or participate in physiotherapy, thus prolonging MV; moreover, these patients may also require vasopressors and may be more susceptible to *delirium*. Conversely, low doses can lead to patient-ventilator dyssynchrony and agitation.^(54)^ Due to these potential adverse effects, we recommend careful work-ups and close monitoring for patients receiving methadone with routine electrocardiograms, especially if there is concomitant administration of medications that may prolong the QT interval. The administration of methadone should be stopped, and patients should be closely assessed if the QT interval is > 500ms.

### Strengths and limitations

Several strengths should be highlighted in this study. For example, we conducted a broad systematic review of the literature via the Cochrane Handbook and reported the results following the PRISMA guidelines. Moreover, an extensive literature search was conducted utilizing multiple databases, and we also used a preregistered protocol and analytical plan. Furthermore, we evaluated the risk of bias and quality of evidence via three different tools. However, our review also has several limitations. First, we changed the primary outcome of our review after completing abstract screening due to a lack of data on the initially planned outcomes. However, our research strategy was broad and included methadone and ICU as factors (Supplementary Material), and we updated our PROSPERO registration to reflect the implemented changes. We also reported and discussed the outcomes regarding pain, *delirium* and agitation scores. Additionally, the overall GRADE quality of the included studies was low, and most of the studies exhibited a moderate-to-serious risk of bias, with notable heterogeneity being observed in patient populations, interventions, comparators, and outcomes. Most of the studies included patients at risk of opioid withdrawal or chronic opioid use, which further limits any benefits related to methadone. Additionally, more than half of the included patients were reported in abstracts that either did not present detailed methodology or were published as manuscripts, thus eliciting concerns about publication bias. Notably, the same group of authors published two abstracts with different outcomes; however, it is impossible to determine whether these publications included the same patients. Low-quality studies may selectively report positive findings, lack external validity, and either overestimate or underestimate the effect of an intervention. Finally, most of the studies compared methadone to shorter-acting opioids or placebo. Thus, the benefits of methadone may be related to its pharmacokinetics and not to its pharmacodynamics.^(55)^ These limitations pose important obstacles to the generalization of our findings.

### Implication statement

The management of pain in mechanically ventilated patients is challenging. Low-quality evidence suggests that methadone, which is a synthetic opioid used to treat opioid use disorder, opioid withdrawal, and chronic pain, may be associated with better patient-centered outcomes. Further high-quality studies are warranted to validate this conclusion.

## CONCLUSION

Significant limitations in the literature warrant caution regarding the use of methadone in mechanically ventilated patients in the intensive care unit. Most of the included studies demonstrated a low quality of evidence, with a moderate-to-high risk of bias being observed. High-quality, well-designed, and adequately powered studies are needed to rigorously evaluate the potential advantages of methadone in this patient population.

## Supplementary Material



## References

[1] Stein-Parbury J, McKinley S (2000). Patients’ experiences of being in an intensive care unit: a select literature review. Am J Crit Care.

[2] Burry L, Cook D, Herridge M, Devlin JW, Fergusson D, Meade M (2015). SLEAP Investigators; Canadian Critical Care Trials Group. Recall of ICU stay in patients managed with a sedation protocol or a sedation protocol with daily interruption. Crit Care Med.

[3] Devlin JW, Skrobik Y, Gélinas C, Needham DM, Slooter AJ, Pandharipande PP (2018). Executive Summary: Clinical practice guidelines for the prevention and management of pain, agitation/sedation, delirium, immobility, and sleep disruption in adult patients in the ICU. Crit Care Med.

[4] Pham T, Heunks L, Bellani G, Madotto F, Aragao I, Beduneau G (2023). WEAN SAFE Investigators. Weaning from mechanical ventilation in intensive care units across 50 countries (WEAN SAFE): a multicentre, prospective, observational cohort study. Lancet Respir Med.

[5] Shehabi Y, Howe BD, Bellomo R, Arabi YM, Bailey M, Bass FE (2019). ANZICS Clinical Trials Group and the SPICE III Investigators. Early sedation with dexmedetomidine in critically ill patients. N Engl J Med.

[6] Wunsch H, Kahn JM, Kramer AA, Rubenfeld GD (2009). Use of intravenous infusion sedation among mechanically ventilated patients in the United States. Crit Care Med.

[7] Elliott K, Kest B, Man A, Kao B, Inturrisi CE (1995). N-methyl-D-aspartate (NMDA) receptors, mu and kappa opioid tolerance, and perspectives on new analgesic drug development. Neuropsychopharmacology.

[8] Murphy GS, Szokol JW, Avram MJ, Greenberg SB, Shear TD, Deshur MA (2017). Clinical effectiveness and safety of intraoperative methadone in patients undergoing posterior spinal fusion surgery: a randomized, double-blinded, controlled trial. Anesthesiology.

[9] Wanzuita R, Poli-de-Figueiredo LF, Pfuetzenreiter F, Cavalcanti AB, Westphal GA (2012). Replacement of fentanyl infusion by enteral methadone decreases the weaning time from mechanical ventilation: a randomized controlled trial. Crit Care.

[10] Machado FC, Vieira JE, de Orange FA, Ashmawi HA (2019). Intraoperative methadone reduces pain and opioid consumption in acute postoperative pain: a systematic review and meta-analysis. Anesth Analg.

[11] Nunn KP, Velazquez AA, Bebawy JF, Ma K, Sinedino BE, Goel A (2025). Perioperative methadone for spine surgery: a scoping review. J Neurosurg Anesthesiol.

[12] Higgins JP, Green S (2008). Cochrane Handbook for Systematic Reviews of Interventions.

[13] Payen JF, Bru O, Bosson JL, Lagrasta A, Novel E, Deschaux I (2001). Assessing pain in critically ill sedated patients by using a behavioral pain scale. Crit Care Med.

[14] Sessler CN, Gosnell MS, Grap MJ, Brophy GM, O’Neal PV, Keane KA (2002). The Richmond Agitation-Sedation Scale: validity and reliability in adult intensive care unit patients. Am J Respir Crit Care Med.

[15] Ramsay MA, Savege TM, Simpson BR, Goodwin R (1974). Controlled sedation with alphaxalone-alphadolone. BMJ.

[16] Riker RR, Picard JT, Fraser GL (1999). Prospective evaluation of the Sedation-Agitation Scale for adult critically ill patients. Crit Care Med.

[17] Ely EW, Inouye SK, Bernard GR, Gordon S, Francis J, May L (2001). Delirium in mechanically ventilated patients: validity and reliability of the confusion assessment method for the intensive care unit (CAM-ICU). JAMA.

[18] Page MJ, McKenzie JE, Bossuyt PM (2021). The PRISMA 2020 statement: an updated guideline for reporting systematic reviews. BMJ.

[19] Sterne JA, Savović J, Page MJ, Elbers RG, Blencowe NS, Boutron I (2019). RoB 2: a revised tool for assessing risk of bias in randomised trials. BMJ.

[20] Sterne JA, Hernán MA, Reeves BC, Savović J, Berkman ND, Viswanathan M (2016). ROBINS-I: a tool for assessing risk of bias in non-randomised studies of interventions. BMJ.

[21] Guyatt G, Oxman AD, Akl EA, Kunz R, Vist G, Brozek J (2011). GRADE guidelines: 1. Introduction-GRADE evidence profiles and summary of findings tables. J Clin Epidemiol.

[22] Jones GM, Coffey G, Miller S, Murphy CV, Porter K, Whitmill ML (2011). Impact of early initiation of methadone in trauma patients requiring mechanical ventilation. Crit Care Med.

[23] Wanzuita R, Westphal G, Gonçalves A, Pfuetzenreiter F, Ribeiro AV, Ayres SA (2007). Less fentanyl requirement by enteral methadone decreases mechanical ventilation duration and intensive care unit length of stay. Crit Care.

[24] Zavala S, Wasilewski G, Sharma A, Bulakh O, Fahmy J, Liu YM (2021). 9 Impact of Methadone Initiation in Intubated Burn Patients: A Retrospective Review. J Burn Care Res.

[25] Maghsoudi B, Emami M, Khosravi MB, Zand F, Tabatabaie HR, Masjedi M (2016). Comparison of opium tincture and methadone on pain and agitation control in inhalational opium addicted patients admitted to critical care unit: a pilot study. ESICM LIVES 2016: part two. Intensive Care Med Exp.

[26] Wanzuita R, Westphal G, Pfuetzenreiter F, Ayres S, Cavalcanti A, Figueiredo L (2011). Replacing fentanyl infusion by enteral methadone decreases weaning time from mechanical ventilation. Crit Care.

[27] Bonnin S, Kim W, Kim A (2020). 976: Enteral methadone use in critically ill patients to decrease continuous opioid infusion requirements. Crit Care Med.

[28] Kowalski K, Droege C, Ernst N, Mueller E, Keegan S, Droege M (2018). 995: Methadone versus scheduled opioid therapy in time to continuous infusion analgesia discontinuation. Crit Care Med.

[29] Jones GM, Porter K, Coffey R, Miller SF, Cook CH, Whitmill ML (2013). Impact of early methadone initiation in critically injured burn patients: a pilot study. J Burn Care Res.

[30] Azimi HA, Keats KR, Sulejmani E, Ortiz K, Waller J, Wayne N (2023). Use of Methadone Versus Oxycodone to Facilitate Weaning of Parenteral Opioids in Critically Ill Adult Patients. Ann Pharmacother.

[31] Al-Qadheeb NS, Roberts RJ, Griffin R, Garpestad E, Ruthazer R, Devlin JW (2012). Impact of enteral methadone on the ability to wean off continuously infused opioids in critically ill, mechanically ventilated adults: a case-control study. Ann Pharmacother.

[32] Najafi B, Shadnia S, Hassanian-Moghaddam H, Heydarian A, Mahdavinejad A, Zamani N (2021). Fentanyl versus methadone in management of withdrawal syndrome in opioid addicted patients; a pilot clinical trial. Arch Acad Emerg Med.

[33] Chiu AW, Contreras S, Mehta S, Korman J, Perreault MM, Williamson DR (2017). Iatrogenic opioid withdrawal in critically ill patients: a review of assessment tools and management. Ann Pharmacother.

[34] Girard TD, Alhazzani W, Kress JP, Ouellette DR, Schmidt GA, Truwit JD (2017). ATS/CHEST Ad Hoc Committee on Liberation from Mechanical Ventilation in Adults. An Official American Thoracic Society/American College of Chest Physicians Clinical Practice Guideline: Liberation from Mechanical Ventilation in Critically Ill Adults. Rehabilitation Protocols, Ventilator Liberation Protocols, and Cuff Leak Tests. Am J Respir Crit Care Med.

[35] Lindberg HB, Steindal SA, Kvande ME (2023;). Critical care nurses’ experiences of caring for patients with iatrogenic opioid withdrawal in the intensive care unit: A qualitative study. Intensive Crit Care Nurs.

[36] Gowing L, Farrell M, Ali R, White JM (2016). Alpha2 -adrenergic agonists for the management of opioid withdrawal. Cochrane Database Syst Rev.

[37] Tobias JD, Schleien CL, Haun SE (1990). Methadone as treatment for iatrogenic narcotic dependency in pediatric intensive care unit patients. Crit Care Med.

[38] Curley MA, Wypij D, Watson RS, Grant MJ, Asaro LA, Cheifetz IM (2015). RESTORE Study Investigators and the Pediatric Acute Lung Injury and Sepsis Investigators Network. Protocolized sedation vs usual care in pediatric patients mechanically ventilated for acute respiratory failure: a randomized clinical trial. JAMA.

[39] Siddappa R, Fletcher JE, Heard AM, Kielma D, Cimino M, Heard CM (2003). Methadone dosage for prevention of opioid withdrawal in children. Paediatr Anaesth.

[40] Lamey PS, Landis DM, Nugent KM (2022). Iatrogenic opioid withdrawal syndromes in adults in intensive care units: a narrative review. J Thorac Dis.

[41] Bowens CD, Thompson JA, Thompson MT, Breitzka RL, Thompson DG, Sheeran PW (2011). A trial of methadone tapering schedules in pediatric intensive care unit patients exposed to prolonged sedative infusions. Pediatr Crit Care Med.

[42] Dervan LA, Yaghmai B, Watson RS, Wolf FM (2017). The use of methadone to facilitate opioid weaning in pediatric critical care patients: a systematic review of the literature and meta-analysis. Paediatr Anaesth.

[43] Hughes MA, Glass PS, Jacobs JR (1992). Context-sensitive half-time in multicompartment pharmacokinetic models for intravenous anesthetic drugs. Anesthesiology.

[44] Smith MT (2000). Neuroexcitatory effects of morphine and hydromorphone: evidence implicating the 3-glucuronide metabolites. Clin Exp Pharmacol Physiol.

[45] Pan S, Anderson TA (2023). Perioperative methadone: perilous or pain panacea?. Anesth Analg.

[46] Martyn JA, Mao J, Bittner EA (2019). Opioid tolerance in critical illness. N Engl J Med.

[47] Hughes CG, Mailloux PT, Devlin JW, Swan JT, Sanders RD, Anzueto A (2021). MENDS2 Study Investigators. Dexmedetomidine or propofol for sedation in mechanically ventilated adults with sepsis. N Engl J Med.

[48] Burry LD, Williamson DR, Perreault MM, Rose L, Cook DJ, Ferguson ND (2014). Analgesic, sedative, antipsychotic, and neuromuscular blocker use in Canadian intensive care units: a prospective, multicentre, observational study. Can J Anesth.

[49] Dale O, Sheffels P, Kharasch ED (2004). Bioavailabilities of rectal and oral methadone in healthy subjects. Br J Clin Pharmacol.

[50] Alinejad S, Kazemi T, Zamani N, Hoffman RS, Mehrpour O (2015). A systematic review of the cardiotoxicity of methadone. EXCLI J.

[51] Martin JA, Campbell A, Killip T, Kotz M, Krantz MJ, Kreek MJ (2011). Substance Abuse and Mental Health Services Administration. QT interval screening in methadone maintenance treatment: report of a SAMHSA expert panel. J Addict Dis.

[52] Anchersen K, Clausen T, Gossop M, Hansteen V, Waal H (2009). Prevalence and clinical relevance of corrected QT interval prolongation during methadone and buprenorphine treatment: a mortality assessment study. Addiction.

[53] Wallner C, Stöllberger C, Hlavin A, Finsterer J, Hager I, Hermann P (2008). Electrocardiographic abnormalities in opiate addicts. Addiction.

[54] Goligher EC, Ferguson ND, Brochard LJ (2016). Clinical challenges in mechanical ventilation. Lancet.

[55] D’Souza RS, Esfahani K, Dunn LK (2023). Pro-Con Debate: role of methadone in enhanced recovery after surgery protocols-superior analgesic or harmful drug?. Anesth Analg.

