# Supplementary material for: Methadone for critically ill patients under mechanical ventilation in the intensive care unit: a systematic review
Source: Crit Care Sci. 2025 Jul 15;37:e20250396. doi: 10.62675/2965-2774.20250396 (PMC12266829; doi:10.62675/2965-2774.20250396)
Supplement: Supplementary file 1 [file 2965-2774-ccsci-37-e20250396-Suppl01.pdf]

Critical Care Science
https://doi.org/10.62675/2965-2774.20250396

SUPPLEMENTARY MATERIAL

Methadone for critically ill patients under mechanical ventilation in the intensive care unit: a systematic review

Sérgio Martins Pereira[1], Megan Abbott[2], João Francisco Figueiredo Marcondes Ferraz[3], Akash Goel[1], Andrea Rigamonti[4], Charmaine de Castro[5], Lisa Burry[6], Airton Leonardo de Oliveira Manoel[7], Michael Chaim Sklar[4]

SEARCH STRATEGY

Keywords used for search strategy

Ovid MEDLINE(R) ALL <1946 to June 22, 2023>

1	Methadone/	13,556
2	(methadone* or 229809935b or 76-99-3 or uc6vbe7v1z or amidone* or biodone* or dolophine* or metadol* or metsedin* or methaddict* or methadose* or methex or phenadone* or phymet or physeptone* or pinadone* or symoron).tw,kf,kw,nm,ot,ox,rx,px.	18,765
3	(synthetic adj1 heroin*).tw,kf,kw.	40
4	or/1-3	18,803
5	Critical Care/	60,375
6	intensive care units/ or burn units/ or coronary care units/ or intensive care units, pediatric/ or recovery room/ or respiratory care units/	87,802
7	(ICU or ICUs or PICU or PICUs or SICU or SICUs or CCU or CCUs).tw,kf,kw.	95,864
8	((intensive or critical or acute) adj3 care).tw,kf,kw.	261,589
9	((cardiac or coronary or heart) adj3 (unit* or centre* or center*)).tw,kf,kw.	16,290
10	(respiratory adj3 (unit* or centre* or center*)).tw,kf,kw.	4,576
11	((surgical or surger*) adj3 (unit* or centre* or center*)).tw,kf,kw.	29,123
12	(burn adj3 (unit* or centre* or center*)).tw,kf,kw.	5049
13	or/5-12	366918
14	4 and 13	291

Embase Classic+Embase <1947 to 2023 June 21>

1	methadone/	38,945
2	(methadone* or 229809935b or 76-99-3 or uc6vbe7v1z or amidone* or biodone* or dolophine* or metadol* or metsedin* or methaddict* or methadose* or methex or phenadone* or phymet or physeptone* or pinadone* or symoron).tw,kf,kw,rn,du,tn,dy.	41,942
3	(synthetic adj1 heroin*).tw,kf,kw.	48
4	or/1-3	41,988
5	intensive care/	148,538

Continue...

This is an open access article under the CC BY license (https://creativecommons.org/licenses/by/4.0/).

...continuation

6	intensive care unit/	226,139
7	burn unit/	3,220
8	coronary care unit/	14,748
9	pediatric intensive care unit/	14,095
10	recovery room/	9,211
11	(ICU or ICUs or PICU or PICUs or SICU or SICUs or CCU or CCUs).tw,kf,kw.	193,476
12	((intensive or critical or acute) adj3 care).tw,kf,kw.	401,144
13	((cardiac or coronary or heart) adj3 (unit* or centre* or center*)).tw,kf,kw.	30,421
14	(respiratory adj3 (unit* or centre* or center*)).tw,kf,kw.	8,433
15	((surgical or surger*) adj3 (unit* or centre* or center*)).tw,kf,kw.	44,887
16	(burn adj3 (unit* or centre* or center*)).tw,kf,kw.	7,942
17	or/5-16	671,146
18	4 and 17	1,353

Wiley Cochrane Database of Systematic Reviews and Central Register of Controlled Trials

Date run: 23/06/2023 17:32

1	MeSH descriptor: [Methadone] this term only	1,487
2	(methadone* or amidone* or biodone* or dolophine* or metadol* or metsedin* or methaddict* or methadose* or methex or phenadone* or phymet or physeptone* or pinadone* or symoron):ti,ab,kw	3,275
3	(229809935b or uc6vbe7v1z):ti,ab,kw	0
4	(synthetic Near/1 heroin*):ti,ab,kw	1
5	#1 OR #2 OR #3 OR #4	3,275
6	MeSH descriptor: [Critical Care] this term only	2,265
7	MeSH descriptor: [Intensive Care Units] this term only	3,278
8	MeSH descriptor: [Burn Units] this term only	49
9	MeSH descriptor: [Coronary Care Units] this term only	164
10	MeSH descriptor: [Intensive Care Units, Pediatric] this term only	400
11	MeSH descriptor: [Recovery Room] this term only	353
12	MeSH descriptor: [Respiratory Care Units] this term only	15
13	(ICU or ICUs or PICU or PICUs or SICU or SICUs or CCU or CCUs):ti,ab,kw18846	18,846
14	((intensive near/2 care) or (critical near/2 care) or (acute near/2 care)):ti,ab,kw	34,472
15	((cardiac near/2 unit*) or (coronary near/2 unit*) or (heart near/2 unit*) or (cardiac near/2 center*) or (coronary near/2 center*) or (heart near/2 center*) or (cardiac near/2 centre*) or (coronary near/2 centre*) or (heart near/2 centre*)):ti,ab,kw	2,087
16	((respiratory near/2 unit*) or (respiratory near/2 centre*) or (respiratory near/2 center*)):ti,ab,kw	408
17	((surgical near/2 unit*) or (surgical near/2 centre*) or (surgical near/2 center*) or (surger* near/2 unit*) or (surger* near/2 centre*) or (surger* near/2 center*)):ti,ab,kw	2,573
18	((burn near/2 unit*) or (burn near/2 centre*) or (burn near/2 center*)):ti,ab,kw	373
19	#6 OR #7 OR #8 OR #9 OR #10 OR #11 OR #12 OR #13 OR #14 OR #15 OR #16 OR #17 OR #18	47,159
20	#5 AND #19	69
	[3 Reviews]	
	[66 Trials]	

EBSCO CINAHL complete

Friday, June 23, 2023 3:50:18 PM

	Query	Limiters/expanders	Last run via	Results
S14	S4 AND S13	Expanders - Apply equivalent subjects Search modes - Boolean/Phrase	Interface - EBSCOhost Research Databases Search Screen - Advanced Search Database - CINAHL Complete	162
S13	S5 OR S6 OR S7 OR S8 OR S9 OR S10 OR S11 OR S12	Expanders - Apply equivalent subjects Search modes - Boolean/Phrase	Interface - EBSCOhost Research Databases Search Screen - Advanced Search Database - CINAHL Complete	179,550
S12	TI ((burn N2 unit*) or (burn N2 centre*) or (burn N2 center*)) OR AB ((burn N2 unit*) or (burn N2 centre*) or (burn N2 center*))	Expanders - Apply equivalent subjects Search modes - Boolean/Phrase	Interface - EBSCOhost Research Databases Search Screen - Advanced Search Database - CINAHL Complete	3,808
S11	TI ((surgical N2 unit*) or (surgical N2 centre*) or (surgical N2 center*) or (surger* N2 unit*) or (surger* N2 centre*) or (surger* N2 center*)) OR AB ((surgical N2 unit*) or (surgical N2 centre*) or (surgical N2 center*) or (surger* N2 unit*) or (surger* N2 centre*) or (surger* N2 center*))	Expanders - Apply equivalent subjects Search modes - Boolean/Phrase	Interface - EBSCOhost Research Databases Search Screen - Advanced Search Database - CINAHL Complete	10,689
S10	TI ((respiratory N2 unit*) or (respiratory N2 centre*) or (respiratory N2 center*)) OR AB ((respiratory N2 unit*) or (respiratory N2 centre*) or (respiratory N2 center*))	Expanders - Apply equivalent subjects Search modes - Boolean/Phrase	Interface - EBSCOhost Research Databases Search Screen - Advanced Search Database - CINAHL Complete	1,029
S9	TI ((cardiac N2 unit*) or (coronary N2 unit*) or (heart N2 unit*) or (cardiac N2 center*) or (coronary N2 center*) or (heart N2 center*) or (cardiac N2 centre*) or (coronary N2 centre*) or (heart N2 centre*)) OR AB ((cardiac N2 unit*) or (coronary N2 unit*) or (heart N2 unit*) or (cardiac N2 center*) or (coronary N2 center*) or (heart N2 center*) or (cardiac N2 centre*) or (coronary N2 centre*) or (heart N2 centre*))	Expanders - Apply equivalent subjects Search modes - Boolean/Phrase	Interface - EBSCOhost Research Databases Search Screen - Advanced Search Database - CINAHL Complete	5,334
S8	TI ((intensive N2 care) or (critical N2 care) or (acute N2 care)) OR AB ((intensive N2 care) or (critical N2 care) or (acute N2 care))	Expanders - Apply equivalent subjects Search modes - Boolean/Phrase	Interface - EBSCOhost Research Databases Search Screen - Advanced Search Database - CINAHL Complete	125,946
S7	TI (ICU or ICUs or PICU or PICUs or SICU or SICUs or CCU or CCUs) OR AB (ICU or ICUs or PICU or PICUs or SICU or SICUs or CCU or CCUs)	Expanders - Apply equivalent subjects Search modes - Boolean/Phrase	Interface - EBSCOhost Research Databases Search Screen - Advanced Search Database - CINAHL Complete	43,516
S6	(MH "Intensive Care Units") OR (MH "Intensive Care Units, Pediatric") OR (MH "Burn Units") OR (MH "Coronary Care Units") OR (MH "Post Anesthesia Care Units") OR (MH "Respiratory Care Units")	Expanders - Apply equivalent subjects Search modes - Boolean/Phrase	Interface - EBSCOhost Research Databases Search Screen - Advanced Search Database - CINAHL Complete	56,884
S5	(MH "Critical Care")	Expanders - Apply equivalent subjects Search modes - Boolean/Phrase	Interface - EBSCOhost Research Databases Search Screen - Advanced Search Database - CINAHL Complete	25,661
S4	S1 OR S2 OR S3	Expanders - Apply equivalent subjects Search modes - Boolean/Phrase	Interface - EBSCOhost Research Databases Search Screen - Advanced Search Database - CINAHL Complete	7,864
S3	TI synthetic N1 heroin* OR AB synthetic N1 heroin*	Expanders - Apply equivalent subjects Search modes - Boolean/Phrase	Interface - EBSCOhost Research Databases Search Screen - Advanced Search Database - CINAHL Complete	55
S2	TI (methadone* or 229809935b or 76-99-3 or uc6vbe7v1z or amidone* or biodone* or dolophine* or metadol* or metsedin* or methaddict* or methadose* or methex or phenadone* or phymet or physeptone* or pinadone* or symoron) OR AB (methadone* or 229809935b or 76-99-3 or uc6vbe7v1z or amidone* or biodone* or dolophine* or metadol* or metsedin* or methaddict* or methadose* or methex or phenadone* or phymet or physeptone* or pinadone* or symoron)	Expanders - Apply equivalent subjects Search modes - Boolean/Phrase	Interface - EBSCOhost Research Databases Search Screen - Advanced Search Database - CINAHL Complete	6,286
S1	(MH "Methadone")	Expanders - Apply equivalent subjects Search modes - Boolean/Phrase	Interface - EBSCOhost Research Databases Search Screen - Advanced Search Database - CINAHL Complete	5,773

National Library of Medicine's PubMed (Not including Medline):

Results: 52; Searched on 23 June 2023, @ 11:22AM EST

(((Methadone[MeSH Terms]) OR (methadone*[Title/Abstract] OR 229809935b[Title/Abstract] OR 76-99-3[Title/Abstract] OR uc6vbe7v1z[Title/Abstract] OR amidone*[Title/Abstract] OR biodone*[Title/Abstract] OR dolophine*[Title/Abstract] OR metadol*[Title/Abstract] OR metsedin*[Title/Abstract] OR methaddict*[Title/Abstract] OR methadose*[Title/Abstract] OR methex[Title/Abstract] OR phenadone*[Title/Abstract] OR phymet[Title/Abstract] OR physeptone*[Title/Abstract] OR pinadone*[Title/Abstract] OR symoron[Title/Abstract])) OR ("Synthetic Heroin"[Title/Abstract:~1])) AND ((((((((((((Critical Care[MeSH Terms]) OR (intensive care units[MeSH Terms])) OR (burn units[MeSH Terms])) OR (coronary care units[MeSH Terms])) OR (intensive care units, pediatric[MeSH Terms])) OR (recovery room[MeSH Terms])) OR (respiratory care units[MeSH Terms])) OR (ICU[Title/Abstract] OR ICUs[Title/Abstract] OR PICU[Title/Abstract] OR PICUs[Title/Abstract] OR SICU[Title/Abstract] OR SICUs[Title/Abstract] OR CCU[Title/Abstract] OR CCUs[Title/Abstract])) OR ((intensive care[Title/Abstract]) OR (critical care[Title/Abstract]) OR (acute care[Title/Abstract]))) OR ((cardiac unit*[Title/Abstract]) OR (coronary unit*[Title/Abstract]) OR (heart unit*[Title/Abstract]) OR (cardiac center*[Title/Abstract]) OR (coronary center*[Title/Abstract]) OR (heart center*[Title/Abstract]) OR (cardiac centre*[Title/Abstract]) OR (coronary centre*[Title/Abstract]) OR (heart centre*[Title/Abstract]))) OR ((respiratory unit*[Title/Abstract]) OR (respiratory centre*[Title/Abstract]) OR (respiratory center*[Title/Abstract]))) OR ((surgical unit*[Title/Abstract]) OR (surgical centre*[Title/Abstract]) OR (surgical center*[Title/Abstract]) OR (surger* unit*[Title/Abstract]) OR (surger* centre*[Title/Abstract]) OR (surger* center*[Title/Abstract]))) OR ((burn unit*[Title/Abstract]) OR (burn centre*[Title/Abstract]) OR (burn center*[Title/Abstract])))) AND (pubstatusaheadofprint OR publisher[SB] OR in process[SB] OR pubmednotmedline[SB])

Scopus

Results: 1119; Searched on 23 June 2023, @2:13PM EST

((TITLE-ABS-KEY (methadone)) OR (TITLE-ABS-KEY (methadone* OR 229809935b OR 76-99-3 OR uc6vbe7v1z OR amidone* OR biodone* OR dolophine* OR metadol* OR metsedin* OR methaddict* OR methadose* OR methex OR phenadone* OR phymet OR physeptone* OR pinadone* OR symoron)) OR (TITLE-ABS-KEY (synthetic W/1 heroin*))) AND ((TITLE-ABS-KEY (icu OR icus OR picu OR picus OR sicu OR sicus OR ccu OR ccus)) OR (TITLE-ABS-KEY ((intensive W/2 care) OR (critical W/2 care) OR (acute W/2 care))) OR (TITLE-ABS-KEY ((cardiac W/2 unit*) OR (coronary W/2 unit*) OR (heart W/2 unit*) OR (cardiac W/2 center*) OR (coronary W/2 center*) OR (heart W/2 center*) OR (cardiac W/2 centre*) OR (coronary W/2 centre*) OR (heart W/2 centre*))) OR (TITLE-ABS-KEY ((respiratory W/2 unit*) OR (respiratory W/2 centre*) OR (respiratory W/2 center*))) OR (TITLE-ABS-KEY ((surgical W/2 unit*) OR (surgical W/2 centre*) OR (surgical W/2 center*) OR (surger* W/2 unit*) OR (surger* W/2 centre*) OR (surger* W/2 center*))) OR (TITLE-ABS-KEY ((burn W/2 unit*) OR (burn W/2 centre*) OR (burn W/2 center*)))

LILACS (Latin American and Caribbean Health Sciences Literature) via VHL Regional Portal

((mh:(methadone)) OR (methadone* OR amidone* OR biodone* OR dolophine* OR metadol* OR metsedin* OR methaddict* OR methadose* OR methex OR phenadone* OR phymet OR physeptone* OR pinadone* OR symoron OR synthetic heroin*))
AND
((mh:(critical care)) OR (mh:(Intensive Care Units)) OR (mh:(Burn Units)) OR (mh:(Coronary Care Units)) OR (mh:(Intensive Care Units, Pediatric)) OR (mh:(Recovery Room)) OR (mh:(Respiratory Care Units)) OR (ICU or ICUs or PICU or PICUs or SICU or SICUs or CCU or CCUs) OR ((intensive care) or (critical care) or (acute care) or (cardiac unit*) or (coronary unit*) or (heart unit*) or (cardiac center*) or (coronary center*) or (heart center*) or (cardiac centre*) or (coronary centre*) or (heart centre*) or (respiratory unit*) or (respiratory centre*) or (respiratory center*) or (surgical unit*) or (surgical centre*) or (surgical center*) or (surger* unit*) or (surger* centre*) or (surger* center*) or (burn unit*) or (burn centre*) or (burn center*)))
**Filtered to LILACS database only**
**Results: 11, searched on June 23, 2023 @ 3:22PM EST**

ClinicalTrials.gov

Other Terms: (ICU or Intensive Care or Intensive Care Unit)
Intervention/Treatment: Methadone
Results: 4 (ClinicalTrials.gov)
4 Studies found for: (ICU or Intensive Care or Intensive Care Unit) | Methadone
